# Optimised PDMS Tunnel Devices on MEAs Increase the Probability of Detecting Electrical Activity from Human Stem Cell-Derived Neuronal Networks

**DOI:** 10.3389/fnins.2017.00606

**Published:** 2017-10-31

**Authors:** Maria Toivanen, Anssi Pelkonen, Meeri Mäkinen, Laura Ylä-Outinen, Lassi Sukki, Pasi Kallio, Mervi Ristola, Susanna Narkilahti

**Affiliations:** ^1^NeuroGroup, BioMediTech Institute and Faculty of Medicine and Biosciences, University of Tampere, Tampere, Finland; ^2^Micro and Nanosystems Research Group, BioMediTech Institute and Faculty of Biomedical Sciences and Engineering, Tampere University of Technology, Tampere, Finland

**Keywords:** human pluripotent stem cells, microelectrode array, neuronal network, tunnel device, *in vitro* model

## Abstract

Measurement of the activity of human pluripotent stem cell (hPSC)-derived neuronal networks with microelectrode arrays (MEAs) plays an important role in functional *in vitro* brain modelling and in neurotoxicological screening. The previously reported hPSC-derived neuronal networks do not, however, exhibit repeatable, stable functional network characteristics similar to rodent cortical cultures, making the interpretation of results difficult. In earlier studies, microtunnels have been used both to control and guide cell growth and amplify the axonal signals of rodent neurons. The aim of the current study was to develop tunnel devices that would facilitate signalling and/or signal detection in entire hPSC-derived neuronal networks containing not only axons, but also somata and dendrites. Therefore, MEA-compatible polydimethylsiloxane (PDMS) tunnel devices with 8 different dimensions were created. The hPSC-derived neurons were cultured in the tunnel devices on MEAs, and the spontaneous electrical activity of the networks was measured for 5 weeks. Although the tunnel devices improved the signal-to-noise ratio only by 1.3-fold at best, they significantly increased the percentage of electrodes detecting neuronal activity (52–100%) compared with the controls (27%). Significantly higher spike and burst counts were also obtained using the tunnel devices. Neuronal networks inside the tunnels were amenable to pharmacological manipulation. The results suggest that tunnel devices encompassing the entire neuronal network can increase the measured spontaneous activity in hPSC-derived neuronal networks on MEAs. Therefore, they can increase the efficiency of functional studies of hPSC-derived networks on MEAs.

## Introduction

Analysis of neuronal network activity *in vitro* is a pivotal part of modern brain disease modelling, neuropharmacological testing, and neurotoxicological screening (Johnstone et al., 2010; Valdivia et al., 2014). *In vitro* neuronal networks derived from human pluripotent stem cells (hPSCs) can replace animal-derived models and better predict responses in humans (Cavanaugh et al., 2014; Hunsberger et al., 2015; Pei et al., 2016). Furthermore, their activity can be measured using microelectrode arrays (MEAs) (Johnstone et al., 2010; Jones et al., 2011; Moser, 2011). For example, the effect of a neurotoxin on the MEA-activity of a hPSC-derived network can be observed before any morphological changes (Ylä-Outinen et al., 2010). However, analyses of MEA data from hPSC-derived networks can be very challenging due to low percentage of electrodes (often <20%), or entire arrays, that detect neuronal activity (Ylä-Outinen et al., 2010; Tukker et al., 2016). Even when the hPSC-derived networks produce robust, measurable activity, the necessary differentiation and functional development can be very slow, taking up to several months (Odawara et al., 2016). The variable and slow development of neuronal network activity on MEA appears to be characteristic to all neuronal cultures of human origin; in primary human neurons, which do not require pre-differentiation, the emergence of electrical activity can take nearly 40 days, where as in corresponding rat neurons the same development happens in only 10 days (Napoli and Obeid, 2016). Therefore, it is clear that new approaches are needed to facilitate the analysis of hPSC-derived neuronal network functions.

Microengineered polydimethylsiloxane (PDMS) devices can be used to answer specific questions on mechanisms of neural function and pathology (Taylor et al., 2005; Scott et al., 2013; Ren et al., 2015), and they can also be used to facilitate the analysis of electrical function of hPSC-derived networks. PDMS devices consisting an open chamber (or “well”) can guide the network to grow more densely on top of the measuring electrodes, and thus facilitate the development and detection of neuronal activity (Kreutzer et al., 2012). PDMS microtunnel devices, on the other hand, increase the detected activity by amplifying extracellular electrical signals detected by the MEA (FitzGerald et al., 2009; Wieringa et al., 2010; Wang et al., 2012). According to a generally accepted theory in the field this occurs because signal amplitudes measured by MEA in tunnels are influenced by a derivative of Ohm's law (U = R I), where the resistance of the medium inside the tunnel increases as the tunnels height (h) and width (w), i.e., cross section (A) decreases and length (l) increases (R = ρ (l / A)). The increased resistance, in turn, manifests as higher potential differences during electrical activity of the measured cells, which translates to an increased signal-to-noise ratio (SNR) in the MEA recordings. However, the microtunnels providing the best amplification (cross sections ≤ 100 μm^2^, *h* ≤ 5 μm) are designed to encompass only neurites and not neuronal somata or entire neuronal networks, and often require a custom made electrode array (FitzGerald et al., 2008; Dworak and Wheeler, 2009; Hong et al., 2017). These neurite-encompassing microtunnels are useful for analysing certain parameters such as the speed of signal conduction along axons, but there is a need for larger tunnel devices which can provide robust MEA data from entire neuronal networks containing also dendrites and cell somata.

In this study, the objective was to develop tunnel devices that are compatible with a commercially available MEA platform, and are able to house entire hPSC-derived neuronal networks and concomitantly possess sufficiently small features to amplify the extracellular signals on MEAs in comparison to standard cultures. Therefore, hPSC-derived neuronal networks were cultured on MEAs in tunnel devices with different dimensions. The spontaneous electrical activity of the neuronal networks in the tunnels was measured up to 5 weeks and compared to data from standard MEA controls. We observed that while the tunnels provided little or no improvement of signal detection, they increased the measured network activity considerably. Thus, the use of the tunnel devices solved one of the main problems in studying hPSC-derived networks using MEAs, which is the low percentage of active electrodes.

## Materials and methods

### Production of MEA-compatible PDMS tunnel devices

**Figure 1:**
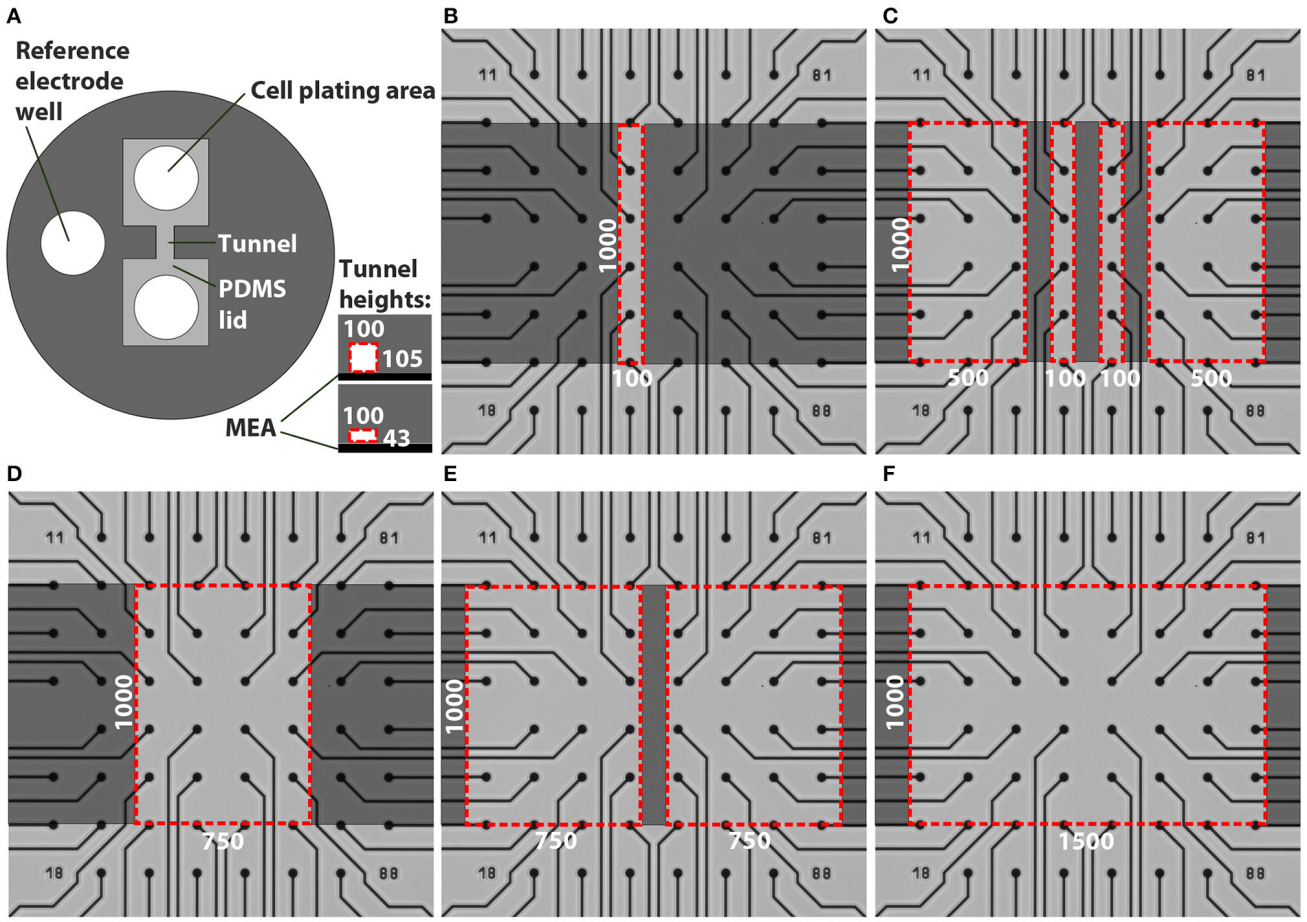
Designs of the tunnel devices. The PDMS tunnel devices **(A)** consist of two cell plating areas that are interconnected by tunnels, and a reference electrode well. The two tunnel heights are presented in the lower right corner in **(A)**. The red dashed lines indicate the cross section of a tunnel, in this case 100 μm wide. The numbers in **(A)** provide the tunnel widths (*w*) and heights (*h*) in μm. Tunnels with different dimensions were aligned on MEAs **(B–F)**. Dark gray indicates PDMS bonded to the MEA surface, light grey represents cell cultivation areas in the tunnels and areas under the PDMS lid, while the red dashed lines indicate tunnel perimeters. The numbers in **(B–F)** provide the tunnel widths (*w*) and lengths (*l*) in μm.

Custom PDMS tunnel devices and SU-8 moulds for the devices were fabricated using rapid prototyping methods (Duffy et al., 1998). The outer diameter of the PDMS devices (Figure 1A) was 15 mm and the height was approximately 3 mm to be compatible with the MEA array (60MEA200/30iR-Ti MEAs, MultiChannelSystem [MCS], Germany) and amplifier (MEA2100, MCS). The PDMS devices contained two cell plating areas that were interconnected by tunnels and a reference electrode well. The designed tunnel dimensions were varied as presented in Table 1 and Figure 1. The cell cultivation area in front of the tunnels is covered by a PDMS lid. The different PDMS tunnels were aligned on top of the MEA electrodes as illustrated in Figures 1B–F.

**Table 1:** Tunnel dimensions and number of tunnels, electrodes and MEAs.

***w* (μm)**	***h* (μm)**	***l* (μm)**	**Number of MEA plates**	**Electrodes per tunnel, outside area or MEA well**	**Total *n* of analysed tunnels, outside areas or MEA wells**	**Total *n* of analysed electrodes**	**Total** ***n*** **of active electrodes per week**				
							**1**	**2**	**3**	**4**	**5**
6-well MEA control			5\–	9	30\–\–	270\–	75	107	120	146	101
1-well MEA control			4\–	59	4\–\–	236\–	–	1	18	39	42
Outside	43	–	13\8	12	13\8\–	156\96	29	87	112	119	118
Outside	105	–	21\15	12	21\15\–	254\180	9	74	150	157	154
100	43	1,000	5\4	4	6\4\5	24\16	13	18	24	24	24
100	105	1,000	7\5	4	9\5\7	36\20	2	14	24	34	31
500	43	1,000	1\–	11–12	2\–\2	23\–	3	2	12	21	23
500	105	1,000	2\–	11–12	4\–\3	46\–	1	6	34	40	44
750	43	1,000	3\2	15–16	4\2\2	63/32	8	24	40	47	48
750	105	1,000	7\5	15–16	9\5\6	142/80	7	48	71	92	91
1,500	43	1,000	5\2	31	5\2\4	155/62	11	69	127	131	127
1,500	105	1,000	7\5	31	7\5\3	217\155	4	39	167	111	137

*The tunnel width (w), height (h) and length (l) are presented in the first three columns. “Outside” refers to electrodes outside the actual tunnels but underneath the PDMS lid (Figure 1). The second number after the backslash indicates the corresponding number in the pharmacological experiment (Figure 6), and the third number after the second backslash indicates n in the CytoSpectre network orientation analysis (Figure 3E)*.

PDMS (Sylgard 184, Dow Corning) devices were fabricated using methods described by Park et al. (2006). The moulds were fabricated from SU-8 3050 (Micro Resist Technology GmbH) on top of a silicon wafer. A 15-mm-diameter punching tool was used to punch individual devices out of PDMS sheets. A 3-mm-diameter manual punching tool was used to create inlets (cell plating areas; Figure 1A) at a distance of 100–1,000 μm from the tunnel mouth, and an opening for the reference electrode. The use of circular cell supply inlets and the need to have equal tunnel lengths created a lid around the punching hole. Thus, the MEA electrodes placed in front of the tunnels were covered by the PDMS lid located either 43 or 105 μm above the MEA surface depending on the tunnel height. Hereafter, these electrodes located underneath the PDMS lid but outside the tunnels are referred as outside electrodes.

The dimensions of the tunnel devices were characterised using both light microscopy and profilometry. A Zeiss Axio Imager.A1m (Carl Zeiss AG) was used to inspect the mould for potential faults. A Bruker Dektak XT stylus profilometer (Bruker Corporation) was used to measure the heights of the microstructures from the mould. According to the measurements, the mould heights were 43 ± 7 μm and 105 ± 15 μm. The variation in heights was caused by the slight bending of the silicon wafers by the spinner vacuum during spin-coating, which caused the features to be thicker in the middle of the moulds.

### Preparation of PDMS tunnel devices and MEAs for cell culture

The MEAs were always cleaned before use according to manufacturer's instructions (washed with 1% Tergazyme [Sigma-Aldrich], rinsed with distilled H_2_O and autoclaved). MEAs were coated with 0.05% polyethylenimine as previously described (Ylä-Outinen et al., 2010). To make the tunnels hydrophilic and thus amenable to coating, the PDMS devices were treated with oxygen plasma in a PICO plasma system (Diener electronic) for 3 min at 50 W. They were manually aligned under a microscope on the MEA electrodes and reversibly bonded to the MEAs, i.e., they could still be manually removed. Mouse laminin (20 μg/ml; Sigma-Aldrich) was pipetted into the PDMS tunnel devices on MEAs through both cell plating areas (Figure 1A). Cell culture control plates (4-well plate, Nunc, Thermo Fisher Scientific, Inc.) were coated with 20 μg/ml or 10 μg/ml mouse laminin in wells with or without coverslips (Ø = 13 mm, VWR), respectively. The MEAs and the control plates were incubated with the laminin solutions at +4°C overnight as previously described (Ylä-Outinen et al., 2010).

### Neural differentiation and cell culture

The human embryonic stem cell (hESC) line Regea 08/023 and the human induced pluripotent stem cell (hiPSC) line 04311.WT were used in the experiments. BioMediTech has approval from the Finnish Medicines Agency (FIMEA) to perform research with human embryos (Dnro 1426/32/300/05). There are also supportive statements from the regional ethical committee of Pirkanmaa Hospital District for the derivation, culturing, and differentiation of hESCs (R05116) and hiPSCs (R08070). This study was carried out in accordance with the recommendations of FIMEA and Pirkanmaa Hospital District with written informed consent from all subjects who provided cell material. All subjects gave written informed consent in accordance with the Declaration of Helsinki.

**Figure 2:**
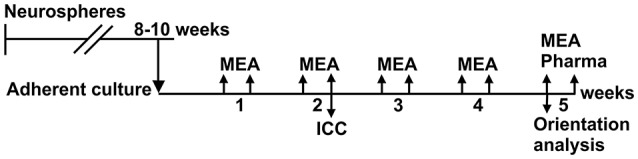
Timeline of the experiments. Neurons were differentiated in neurosphere culture for 8–10 weeks before adherent culture in tunnel devices on MEAs and in standard cell culture wells (control cells). MEA measurements were performed twice weekly over 5 weeks, and a pharmacological test was performed at the end of the culture. Neurite orientation analysis was performed from the phase contrast images taken at the 5th week. Immunocytochemistry was performed on control cells after 2 weeks in adherent culture for both cell lines.

The timeline for the experiments is shown in Figure 2. The hESCs and hiPSCs were differentiated into neural cells for 8–10 weeks in neurosphere cultures in differentiation medium (NDM) consisting of 1:1 Dulbecco's Modified Eagle's Medium/F12:Neurobasal Medium supplemented with 2 mM GlutaMax, 1x B27 supplement, 1x N2 supplement (all from Gibco Invitrogen), 25 U/ml penicillin/streptomycin (Lonza Group Ltd) and, in this neurosphere differentiation stage, 20 ng/ml basic fibroblast growth factor (bFGF, R&D Systems) as previously described (Lappalainen et al., 2010) with or without low-dose naltrexone LDN193189 (100 nM; Stemcell Technologies, Inc.).

The neurospheres containing pre-differentiated neural cells were manually dissected into small cell aggregates (Ø~50–200 μm). Approximately 15 small aggregates (50,000–150,000 cells in total) were plated in both cell plating areas of the PDMS devices (i.e., both ends of the tunnels) as close to the tunnels as possible to ensure tunnel colonization, and similarly on control plate wells. The plating procedure was identical for each device. The PDMS devices on the MEAs were submerged in the cell culture medium (1 ml). The cells were maintained in a humidified incubator at 37°C and 5% CO_2_. Half of the medium was changed three times a week. After 1 week in adherent culture, 4 ng/ml bFGF and 5 ng/ml brain-derived neurotrophic factor (BDNF) (Gibco Invitrogen) were added to the medium.

### Immunocytochemistry

The control cells were fixed after 14 days in adherent culture, and immunocytochemical (ICC) staining was performed as previously described (Lappalainen et al., 2010) to verify the neural identity of the cells. Primary antibodies, rabbit anti-beta-III Tubulin (β-tub) (1:2000; GenScript) and rabbit polyclonal anti-Microtubule-Associated Protein 2 (MAP2) (1:400; Millipore), were used together with secondary antibodies, Alexa 488 anti-rabbit and Alexa 568 anti-mouse (both 1:400; Molecular Probes). In addition, the nuclei of the cells were stained with 4′,6-diamidino-2 phenylindole (DAPI), which was included in the mounting medium (Vectashield Mounting Medium with DAPI, Vector Laboratories). The cells were imaged with a fluorescence microscope (Olympus IX51, Olympus Corporation).

### Phase contrast microscopy and neurite orientation analysis

The cells were imaged with a phase contrast microscope (Nikon Eclipse TE2000-S, Nikon Corporation) once per week to follow the neuronal morphology, migration and network formation. The cell culture control plates were used as a normal microenvironment cell control to evaluate neuronal viability, morphology and migration. The control plates were followed for 2 weeks.

The orientation of the neurites was analysed using CytoSpectre 1.2 software (http://www.tut.fi/cytospectre) (Kartasalo et al., 2015). The software utilises spectral analysis and calculates the orientations of image components, in this case neurites (Hyysalo et al., 2017), and describes their variance with a circular variance value, which is 1 when the components are randomly aligned, and 0 when all components are completely unidirectional. The software was used in the mixed component mode and spectral resolution/noise was set to balanced. Wavelength settings (component size) were set to 1 μm (minimum) and 30 μm (maximum). The orientation of neurites inside the tunnels was analysed using phase contrast images obtained at week 5 after plating, and compared to images of the freely growing networks on cell culture control plates. The electrodes and their tracks were excluded from the image analysis by using a custom MATLAB script which automatically detected the electrodes and the tracks and replaced their pixel values with local mean intensities computed from the corresponding regions of each image, and by using CytoSpectre's component size filtering.

### MEA measurements

MEA measurements were performed with an MEA system consisting of a filter amplifier MEA2100, software MC_Rack and temperature controllers TC02 set at 37°C (all from MCS). The electrical activity of the neuronal networks was measured twice a week for 5 weeks (Figure 2). The duration of each recording was 10 min and the sampling rate was 25 kHz. To analyse the noise, signal amplitude and SNR in the tunnel devices, the MEA data were compared to earlier recordings from the same 60MEA200/30iR-Ti MEAs with no PDMS tunnel devices, referred to as the 1-well MEA control. The 1-well MEA control is an open volume system with no liquid volume restrictions. To analyse the development of network activity, the data were compared to recordings from 60-6wellMEA200/30iR-Ti MEAs (MCS), in which the individual wells were separated using SpikeBooster devices (BioMediTech) (Kreutzer et al., 2012), and is referred to as the 6-well MEA control. The dimensions of the cell culturing areas on the SpikeBooster devices are the same as the cell plating areas on the tunnel devices, and the 6-well MEA control can be considered a partially restricted volume system. The combination of 6-well MEAs and SpikeBooster is the most used MEA setup in our laboratory, and it typically provides the best network activity development. All used MEAs had the same surface material (Si_3_N_4_), electrode material (TiN), electrode diameter (30 μm) and electrode-to-electrode distance (200 μm).

Pharmacological testing with tetrodotoxin (TTX; 1 μM, Tocris Bioscience) was performed at the end of the study (Figure 2). MEA activity was measured for 5 min after addition of fresh medium to the MEA and after addition of TTX to the medium where the PDMS device was submerged. TTX and the equipment used for handling it were stored, handled and disposed according to institutional safety regulations (BioMediTech institute and Faculty of Medicine and Biosciences, University of Tampere).

### Signal analysis and statistics

Spikes were detected from the MEA data using MATLAB (The MathWorks, Inc.) with a custom-made analysis program based on Quiroga et al. (2004). Analysis was performed separately for each electrode (modified from Quiroga et al., 2004). First, the voltage signal was filtered (200–3,000 Hz band pass). Next, the noise was calculated as the median (md) of the absolute values from the filtered recording divided by 0.6745. Signal values which exceeded five times this noise value were considered as spikes. Both negative and positive spikes were detected. Spikes larger than 500 times noise were removed as artefacts. For spike waveform analysis, 0.8 and 1.76 ms of voltage signal was clipped before and after the largest absolute value of the spike from the filtered data. The detector dead time between two waveforms was 1.48 ms. The peak-to-peak amplitudes were measured as the difference between the highest and lowest voltage values in the stored waveforms. A peak-to-peak md was obtained from all waveforms from one channel to identify a single value per channel. SNR was calculated by dividing the md peak-to-peak spike amplitudes by the corresponding noise values. An electrode was regarded as an active electrode (measuring neuronal activity) if more than 2 spikes were recorded in a minute (spike frequency 0.033 Hz). The threshold was determined by measuring the spike rates from empty MEAs and MEAs with TTX-silenced neuronal cultures (data not shown). Percentage of active electrodes was calculated for each tunnel and control well separately and electrodes underneath the PDMS devices were excluded from the analysis. Data from the electrodes at the tunnels mouth (under the red dashed line in Figures 1B–F) were not included in analyses because they could be considered neither outside nor tunnel electrodes. Bursts (clusters of spikes) were detected separately for each electrode using a method based on Kapucu et al. (2012) which defines bursts using the cumulative moving average of inter-spike intervals.

The number of repeats (*n*) in different analyses are presented in Table 1. Statistical analyses were performed in SPSS (IBM). The MEA data were found to have a non-normal distribution, and therefore the nonparametric Kruskal-Wallis test with Dunn's *post hoc* test was used to determine whether there were statistically significant differences among the different tunnels and controls. The data from the neurite orientation analysis (CytoSpectre results) were found to be normally distributed and thus were analysed by univariate analysis of variance with Bonferroni's *post hoc* test. A *p*-value less than 0.05 was considered significant.

## Results

### Neuronal network cultures in tunnel devices

**Figure 3:**
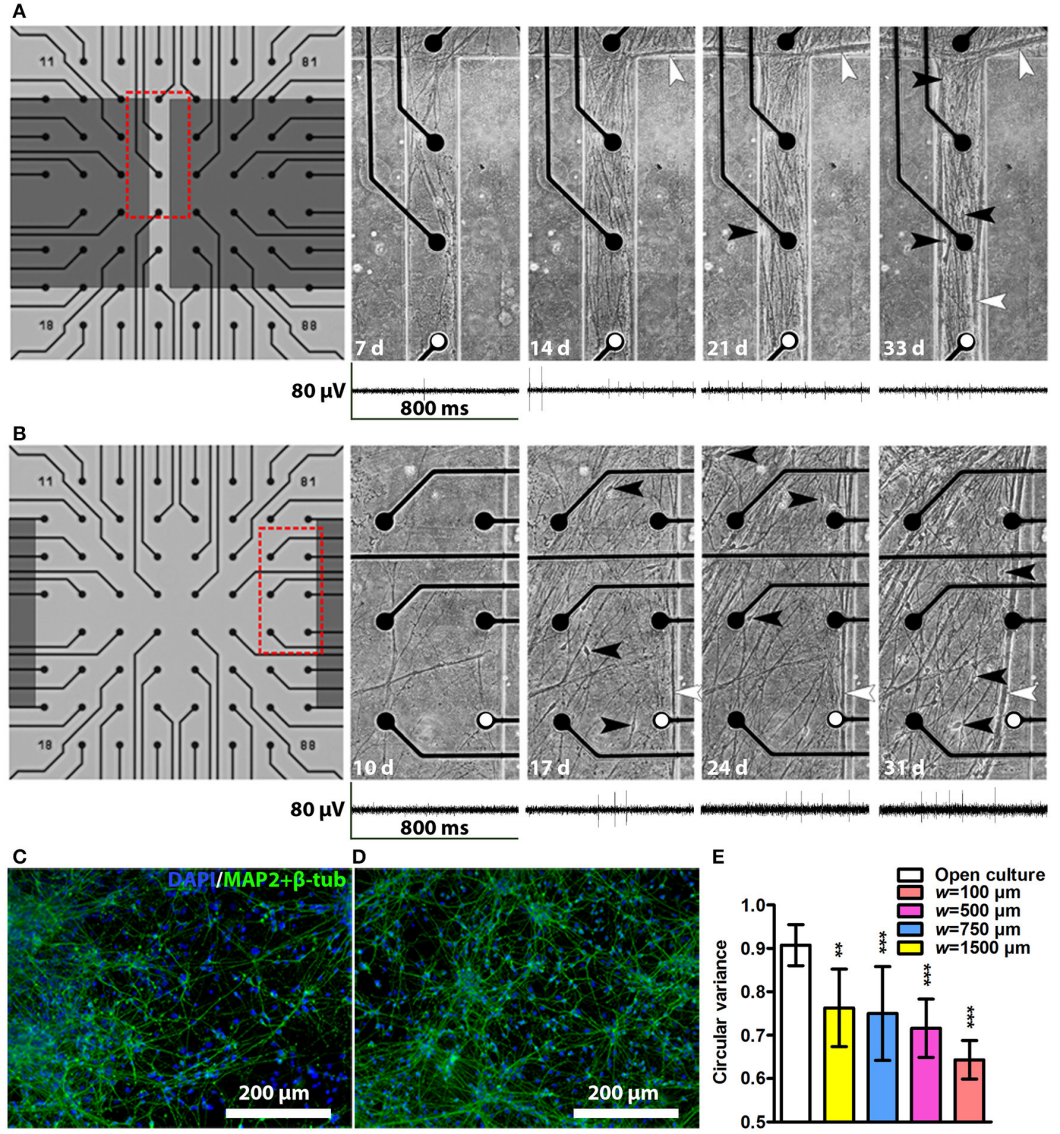
Network development in tunnels and immunocytochemistry images of neuronal cultures. Network development in an *h* = 43 μm, *w* = 100 μm tunnel **(A)** and an *h* = 43 μm, *w* = 1,500 μm tunnel **(B)**. The electrode-to-electrode distance is 200 μm. Red dashed boxes in **(A,B)** show the location of the corresponding subfigures on the right, where somata are indicated by black and neurite bundles by white arrowheads, d, days. Representative MEA traces from the same week are shown under each tunnel image. The electrodes from which the traces were obtained are marked with white dots. Immunocytochemistry images of two cell lines: **(C)** the hiPSC line 04311.WT and **(D)** the hESC line 08/023 growing on cell culture controls plates. The cell nuclei (DAPI, blue) and neuronal markers (microtubule-associated protein 2 [MAP2] and class III tubulin [β-tub], green) were stained. **(E)** Neurite orientation analysis confirmed significantly smaller circular variances of the networks in tunnels than in freely growing open cultures. *n*_open culture_ is 8 and *n* otherwise is 2–7 (Table 1). Statistical difference between the groups was analysed using univariate analysis of variance, and the ^*^ symbols indicate significance based on Bonferroni's *post hoc* test vs. freely growing open cultures (^*^0.05 > *p* ≥ 0.01; ^**^0.01 > *p* ≥ 0.001; ^***^p > 0.001).

After cell plating, the neurons started to migrate and elongate neurites into the tunnels. The first neurites and neurons entered the tunnels as early as 3 days after plating, and typically by 2–3 weeks the neurons had formed a network covering approximately the entire area inside the tunnels. Examples of network growth from the narrowest (*w* = 100 μm) and widest tunnels (*w* = 1,500 μm) are shown in Figures 3A,B, respectively. Occasionally, the reversible PDMS-MEA bonding led to partial detachment of some of the PDMS devices from the MEAs. The cultures with insufficient PDMS-MEA bonding were excluded from the experiments. The neuronal nature of the used cells was verified by immunocytochemical staining for known neuronal markers (Figures 3C,D). Cell viability in the tunnels was good, and no significant cell death or detachment was observed with phase contrast microscopy during the 5-week culture period. The tunnels contained neurites and cell somata migrated into the tunnels. Toward the end of the culture the neurites tended to form thick bundles that were typically next to the PDMS walls regardless of the tunnel width. All tunnels affected neuronal network development by causing neurite orientation (Figures 3A,B) compared with random neuronal networks without tunnels (Figures 3C,D). This observation was verified by the neurite orientation analysis, which showed that the mean circular variance values in the networks inside tunnels were significantly smaller than in freely growing networks [*F*_(3, 31)_ = 6.63, *p* = 0.001; Figure 3E]. The tunnel height had no significant effect on the neurite orientation [*F*_(1, 31)_ = 2.94, *p* = 0.097]. The neurites in the narrowest (*w* = 100 μm) tunnels were the most unidirectional (Figures 3A,E,).

### MEA signal detection inside tunnel devices

**Figure 4:**
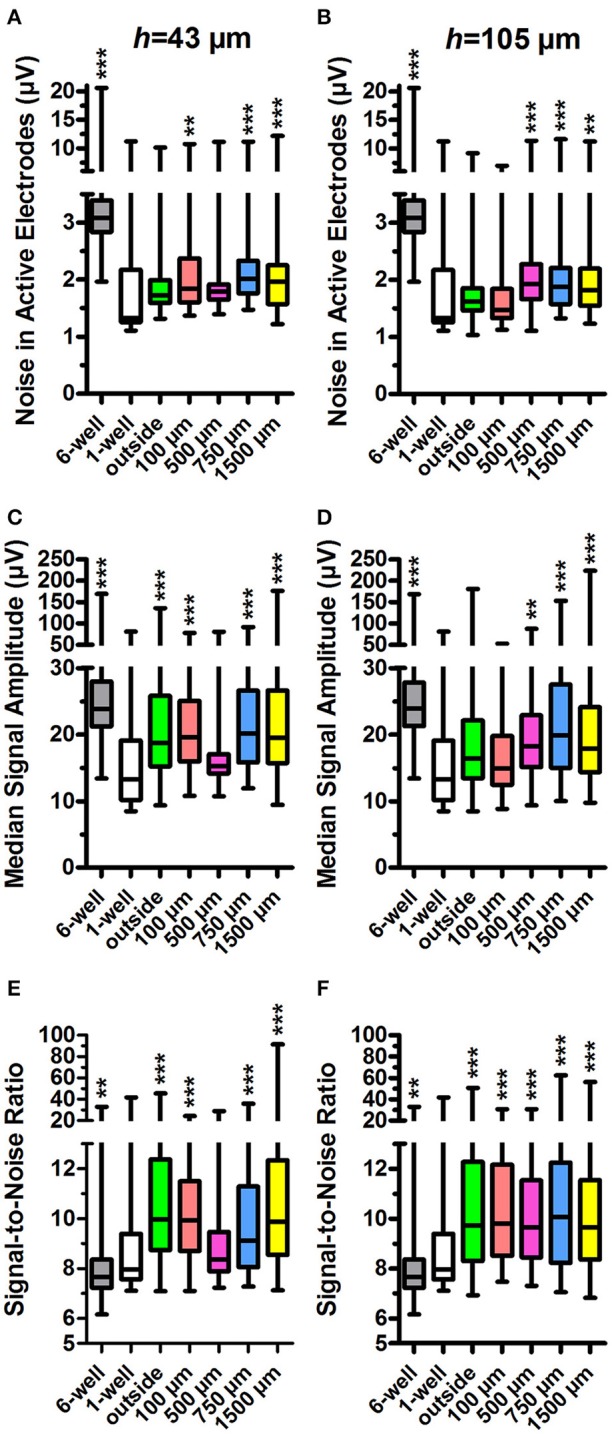
Noise in active electrodes, signal amplitude and signal-to-noise ratio. The noise was calculated for active electrodes in *h* = 43 μm **(A)** and *h* = 105 μm tunnel devices **(B)** and controls (6 and 1-well MEAs). The tunnel width or control group is indicated on the x-axes. The median signal amplitude in active electrodes in *h* = 43 μm **(C)** and *h* = 105 μm devices **(D)** and controls was also calculated. The signal-to-noise-ratio (SNR) in *h* = 43 μm **(E)** and *h* = 105 μm devices **(F)** was calculated from the noise and median signal amplitude in active electrodes. *n* (active electrodes) is 61–547 (weekly numbers of active electrodes are in Table 1). Please note that the low number of *h* = 43 μm, *w* = 500 μm tunnels (2) may affect the results. Statistical differences between groups were analysed using the Kruskal-Wallis test, and the ^*^ symbols indicate significant differences using Dunn's *post hoc* test vs. the 1-well control (^*^0.05 > *p* ≥ 0.01; ^**^0.01 > *p* ≥ 0.001; ^***^p > 0.001).

To determine whether the different tunnel dimensions affected the signal detection on MEA, we calculated the noise values, md peak-to-peak signal amplitudes, and from these, the corresponding SNRs in each active electrode using a custom-made MATLAB algorithm (Figure 4). Examples of the neuronal signals are shown in the narrow tunnel (*w* = 100 μm) and wide tunnel (*w* = 1,500 μm) (Figures 3A,B).

Data from standard 1-well MEAs served as the relevant control for these parameters because the tunnel device data were obtained using the same individual MEAs. The 1-well MEA control data did not contain measurements from the first week after plating, and week 1 data were therefore omitted from this analysis but are presented in Supplementary Figure 1. The outside-group refers to the electrodes outside the tunnels but under the PDMS lid (Figure 1), and thus have the same *h* as the tunnel electrodes.

The md noise values were higher in the electrodes inside the PDMS tunnels in comparison to the 1-well MEA control (md 1.3 μV; Figures 4A,B). In the *h* = 43 μm tunnels, noise was significantly increased in the electrodes inside *w* = 100 μm, *w* = 750 μm, and *w* = 1,500 μm tunnels (md 1.8, 2.0, and 2.0, *p* < 0.001; Figure 4A). The 6-well MEA control had a comparatively high noise level (md 3.1 μV). In the *h* = 105 μm, the tunnels noise was significantly increased in *w* = 500 μm, *w* = 750 μm, and *w* = 1,500 μm tunnels (md 1.9, 1.9, and 1.8; *p* < 0.001; Figure 4B). In the *h* = 105 μm devices, the noise was particularly high at the first week after plating (Supplementary Figure 1), probably due to system stabilization and/or protein adsorption to the electrode surface. However, inclusion of the data in the analyses had no significant effect on the results. In general, noise was significantly higher in the *h* = 43 μm devices compared with the *h* = 105 μm devices (*p* < 0.001; Figure 4A vs. Figure 4B). These results suggest that PDMS tunnels can increase noise in MEA recordings depending on the tunnel dimensions.

In agreement with the increased noise values, the md peak-to-peak signal amplitudes were increased in electrodes inside tunnel devices when compared with the 1-well MEA control (md 13.3 μV; Figures 4C,D). In the *h* = 43 μm tunnel devices, the amplitudes were significantly increased in the outside electrodes as well as the electrodes in *w* = 100 μm, *w* = 750 μm and *w* = 1,500 μm tunnels (md 18.4, 18.9, 20.1, and 19.5 μV, respectively; *p* < 0.001; Figure 4C). The 6-well MEA control had relatively high signal amplitudes (md 24.3 μV). In the *h* = 105 μm tunnels, the amplitudes were significantly increased in the electrodes in *w* = 500 μm, *w* = 750 μm, and *w* = 1,500 μm tunnels (md 18.2, 20.0, and 18.0 μV; *p* < 0.001; Figure 4D). In general, signal amplitudes were significantly higher in the electrodes in the *h* = 43 μm devices compared with the *h* = 105 μm devices (*p* < 0.001; Figure 4C vs. Figure 4D). These results suggest that PDMS tunnel devices can increase MEA signal amplitude depending on the device dimensions.

SNR was found to be higher in the electrodes in tunnel devices compared with the 1-well MEA control (md 8.0, Figures 4E,F). In the *h* = 43 μm tunnel devices, SNR was significantly increased in the outside electrodes under the PDMS lid as well as electrodes in *w* = 100 μm, *w* = 750 μm, and *w* = 1,500 μm tunnels (md 10.0, 9.9, 9.1, and 9.9, respectively; *p* < 0.001; Figure 4E). SNR was lowest in the 6-well MEA controls (md 7.7). In the *h* = 105 μm tunnel devices, SNR was significantly increased in the outside electrodes as well as electrodes in *w* = 100 μm, *w* = 500 μm, *w* = 750 μm, and *w* = 1,500 μm tunnels (md 9.7, 9.8, 9.6, 10.1, and 9.7, respectively; *p* < 0.001; Figure 4F). The tunnel height had no significant effect on SNR (*p* = 0.244; Figure 4E vs. Figure 4F). In summary, the SNRs recorded from inside the tunnel devices were very similar, thus making the differences in noise and signal amplitudes between different designs irrelevant in terms of MEA signal detection. However, it appears that a PDMS tunnel device on the MEA in general improves SNR.

### Tunnel devices increase spike and burst activity on MEA

**Figure 5:**
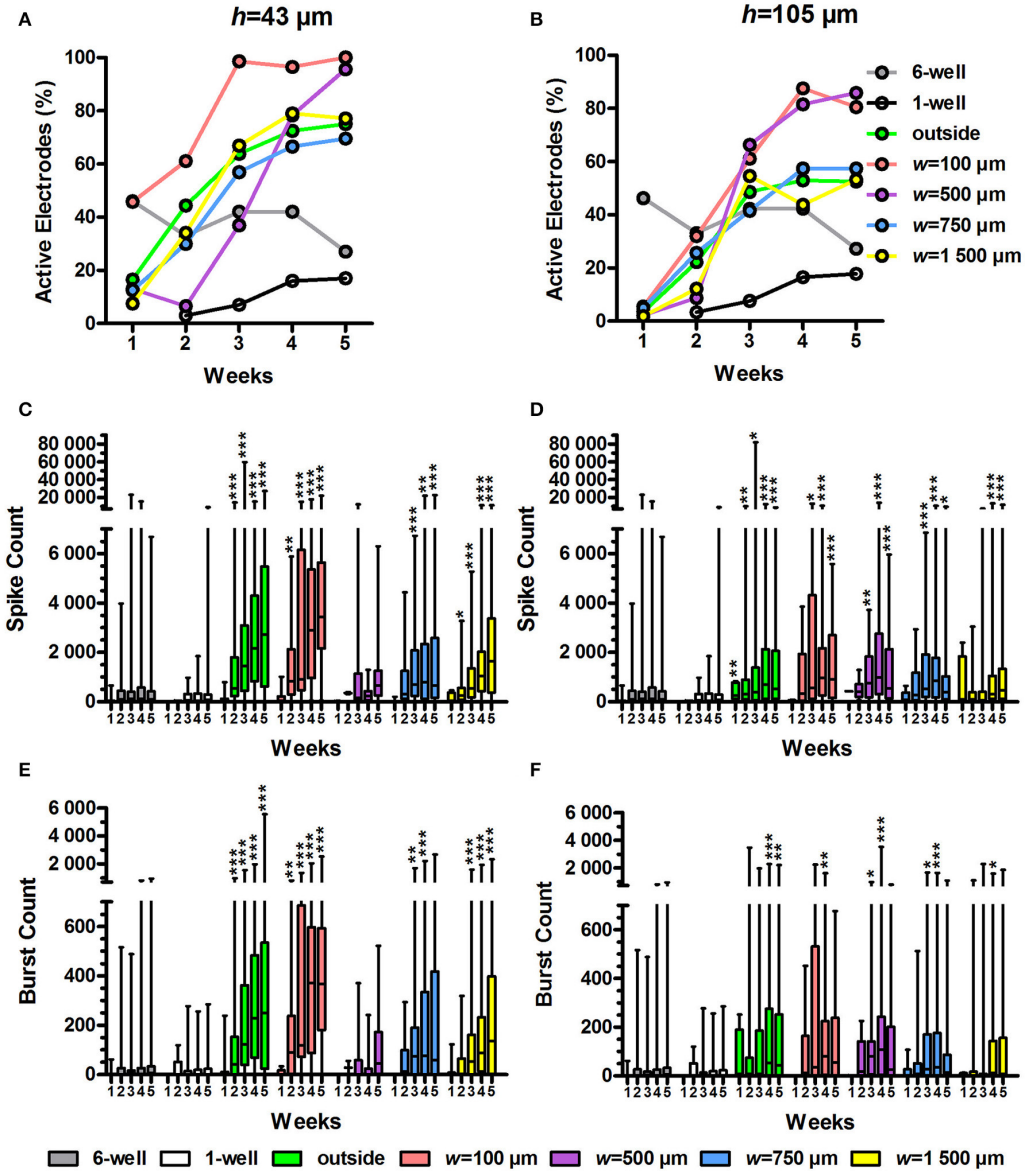
Percentage of active electrodes, spike count and burst count. The percentage of active electrodes was calculated from the MEA data for the *h* = 43 μm **(A)** and *h* = 105 μm tunnel devices **(B)** as well as the controls (6 and 1-well MEAs). Total *n* of analysed electrodes was 23-270 (Table 1). The spike count in the active electrodes over 10 min was also analysed for the *h* = 43 μm **(C)** and *h* = 105 μm tunnel devices **(D)** and controls. The number of bursts over 10 min was analysed from the spike data for the *h* = 43 μm **(E)** and *h* = 105-μm-high tunnel devices **(F)** and the controls. *n* (active electrodes) was 1-167 (Table 1). Please note that the low number of *h* = 43 μm, *w* = 500 μm tunnels (2) may affect the results. Statistical differences between groups each week were analysed using the Kruskal-Wallis test, and ^*^ symbols indicate significant differences using Dunn's *post hoc* test vs. the 6-well control (^*^0.05 > *p* ≥ 0.01; ^**^0.01 > *p* ≥ 0.001; ^***^p > 0.001).

To assess whether the tunnel devices could affect the spike and burst activity on MEA, we analysed the percentage of active electrodes, spike count and burst count in active electrodes using our custom-made MATLAB algorithm (Figure 5). We compared the activity data from tunnel electrodes to the 6-well MEA control. The statistical analyses between the tunnel devices and controls were performed separately each week because the activity in the tunnel electrodes increased dramatically over time.

The percentage of active electrodes increased in the tunnel devices (Figures 5A,B). At week 1, the percentage of active electrodes was highest in the 6-well MEA controls. However, while the percentage in the 6-well MEA controls decreased from 46 to 27% over 5 weeks, the percentage in the tunnel devices steadily increased, surpassing the 6-well MEA controls 2–4 weeks after plating and finally reaching 52–100% at week 5. The percentage of active electrodes was especially high in the *w* = 100 μm and *w* = 500 μm tunnels, reaching 80–100% by week 5. The percentages were also higher in the *h* = 43 μm tunnels compared with the *h* = 105 μm tunnels (69–100% vs. 52–85% at week 5; Figure 5A vs. Figure 5B). The smallest tunnels (*h* = 43 μm, *w* = 100 μm) were best in terms of the percentage of active electrodes, reaching 100% as early as week 3 (Figure 5A).

There were significantly more spikes per active electrode in the tunnel devices compared with the 6-well MEA controls (Figures 5C,D). The md spike count in the 6-well MEA controls never reached higher than 125 over 10 min (week 4). The md spike count was highest, with 3,449 spikes over 10 min, in the smallest tunnels (*h* = 43 μm, *w* = 100 μm) at week 5 (*p* < 0.001; Figure 5C). The outside electrodes in the *h* = 43 μm tunnel devices also had high spike counts, with md reaching 2,715 at week 5. The spike counts between the outside electrodes and the *w* = 100 μm tunnels did not differ significantly during any week. The spike counts were also significantly increased in higher (*h* = 105 μm) devices compared with the 6-well MEA controls (Figure 5D). The maximal spike count in the *h* = 105 μm devices was reached at week 4, when the md spike count in the *w* = 500 μm tunnels was 992 (*p* < 0.001). The md spike count was nearly as high in the *w* = 100 μm tunnels for the corresponding time point (964). In general, the spike count was higher in the *h* = 43 μm tunnel devices compared with the *h* = 105 μm devices. For example, in the *w* = 100 μm tunnels, the difference was significant at weeks 4 (2,886 and 964, respectively; *p* = 0.013) and 5 (3,449 vs. 907, respectively; *p* = 0.002; Figure 5C vs. Figure 5D). Taken together, the spike count data showed that the *w* = 100 μm tunnels and outside area of the *h* = 43 μm tunnel devices were the best to increase the amount of measured network activity.

As with the spike counts, the burst counts were also increased in the tunnel devices compared with the 6-well MEA controls. In the 6-well MEA controls, the md burst count was highest at week 5, with 6 bursts over 10 min (Figures 5E,F). In the smallest tunnels (*h* = 43 μm, *w* = 100 μm), the burst count reached a maximum, 373, at week 4 (*p* < 0.001; Figure 5E). The burst counts were also very high in the outside electrodes of the *h* = 43 μm tunnel devices, with 250 bursts at week 5 (*p* < 0.001). The burst counts between the outside electrodes and the *w* = 100 μm tunnels did not differ significantly during any week. The burst counts were also significantly increased in the higher (*h* = 105 μm) tunnels compared with the 6-well MEA controls, achieving a maximum of 108 at week 4 in the *w* = 500 μm tunnels (*p* < 0.001; Figure 5F). The md burst count was also high in the *w* = 100 μm tunnels at the same week, with 80 bursts. However, the number of bursts in the *h* = 105 μm tunnels was generally less than in the *h* = 43 μm tunnels. For example, in the *w* = 100 μm tunnels, the difference was significant at weeks 4 (80 and 373, respectively; *p* = 0.006) and 5 (56 and 368, respectively; *p* = 0.002; Figure 5E vs. Figure 5F). The burst count data suggested that the MEA activity was highest in *w* = 100-μm-wide tunnels and the outside area of the *h* = 43-μm-high tunnel devices, which is consistent with the spike count results (Figures 5C,D). However, considering also the percentage of active electrodes (Figures 5A,B), it is not the area outside the tunnels, but particularly the smallest tunnels (*h* = 43 μm, *w* = 100 μm), that had the greatest ability to increase the amount of measured network activity.

### MEA activity inside tunnel devices can be affected by pharmacological treatment

**Figure 6:**
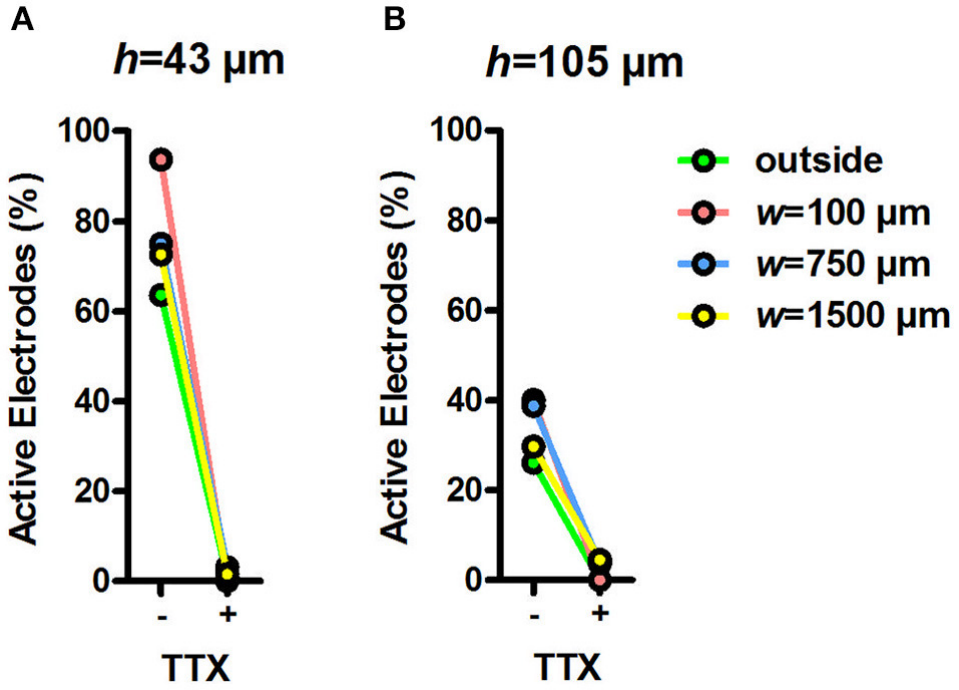
Percentage of active electrodes before and during TTX. Percentage of active electrodes in *h* = 43 μm **(A)** and *h* = 105 μm tunnel devices **(B)** during 5-min recordings after changing the medium and adding TTX. Total *n* of analysed electrodes was 16–180 (Table 1).

To test whether the MEA signals inside the tunnel devices originated from neuronal activity, we measured the MEA activity inside the devices before and after TTX treatment (Figures 6A,B). TTX inhibits the function of neuronal voltage-gated sodium channels and, therefore, blocks the electrical activity of neurons. After addition of regular medium, the percentage of active electrodes was between 26% (*h* = 105 μm, outside electrodes) and 94% (*h* = 43 μm, *w* = 100 μm). After the addition of TTX to the medium, the percentage of active electrodes dropped between 5% (*h* = 105 μm, *w* = 1,500 μm) and 0% (e.g., *h* = 43 μm, *w* = 100 μm). The effect of TTX on MEA activity in tunnel devices was prompt and clear, showing that the measured activity was of neuronal origin and that the tunnel devices can be used in pharmacological experiments.

## Discussion

The aim of this study was to develop novel tunnel devices that would facilitate and enhance the detection and analysis of extracellular electrical signals recorded from hPSC-derived neuronal networks. Neurons were cultured on MEAs covered by PDMS tunnel devices with varying dimensions, and the spontaneous electrical activity was measured for 5 weeks. The tunnels induced unidirectional growth of neurites, and the neuronal networks filled the tunnels in 2–3 weeks. The PDMS tunnel devices in general improved SNR, although none of the tested tunnel devices improved the MEA signal detection more than others. The tunnel devices increased network activity, and the smallest tunnels (*h* = 43 μm, *w* = 100 μm) were the best ones in this regard. The neuronal activity inside the tunnels was affected by TTX treatment.

Previously, tunnel devices on MEAs have been studied using rodent derived networks (Goyal and Nam, 2011), but the current setup is, to the best of our knowledge, the first to test the compatibility of PDMS tunnels with neuronal networks of human origin. Cells were plated to both ends of the tunnels, and this ensured neurite growth and cell migration into the tunnels. The hPSC-derived networks remained viable and active for the entire 5-week follow-up period, and we did not observe viability problems that could result from too small medium volume or flow inside the tunnels. The tunnels induced unidirectional growth of neurites, in contrast to the random orientation in freely growing cultures. Interestingly, similar tunnels in the study of Goyal and Nam (2011) appeared to instead induce more random neurite orientation and to increase branching of rat primary hippocampal neurons. This difference may reflect both the developmental stage of the neurons and their region-specific morphology (Dotti et al., 1988). It is also possible that there is some selectivity in the tunnels for axons over dendrites, which can explain some of the observed neurite orientation. However, the tunnels did not exclude dendrites because plenty of cell somas, from which dendrites branch off, migrated into the tunnels. The two cell populations attracting each other from the opposite ends of the tunnel probably also cause the neurites to grow in a more oriented manner. Overall, the results suggest that hPSC-derived neuronal networks are compatible with PDMS tunnel devices on MEA.

In this study, we aimed to improve the detection of electrical activity from human stem cell-derived neuronal networks on MEA using tunnel devices. A generally accepted theory in the field states that signal amplitudes measured by MEA in tunnels are influenced by a derivative of Ohm's law, where the resistance of the medium inside the tunnel increases as the tunnels cross section (*h* and *w*) decreases and length increases (FitzGerald et al., 2008, 2009; Wang et al., 2012). The increased resistance, in turn, manifests as higher potential differences during electrical activity of the measured cells, i.e., higher signal amplitudes measured by the MEA. However, apart for the slightly higher signal amplitudes in the *h* = 43 μm in comparison to the *h* = 105 μm tunnels, there was no clear connection between tunnel the cross section (*h* and *w*) and the signal amplitudes. Additionally, earlier studies examining tunnel devices have reported significant, considerably larger increases in noise and signal amplitudes when measuring rodent neurons (Morales et al., 2008; Dworak and Wheeler, 2009; FitzGerald et al., 2009; Goyal and Nam, 2011; Habibey et al., 2015). The *w* and *h* of our smallest tunnels (*w* = 100 μm, *h* = 43 μm) were most similar to the tunnels described by Goyal and Nam (*w* = 50 or 100 μm, *h* = 50 μm) (Goyal and Nam 2011), who obtained spike amplitudes of several hundred μV, considerably higher than in our tunnels. This difference is most likely due to different tunnel lengths, since the length of the tunnels described by Goyal and Nam was 7-fold compared with our tunnels, and such an increase in the tunnel length has been shown to amplify the signal significantly (FitzGerald et al., 2008, 2009). However, in terms of signal detection, it is most useful to study the ratio between the noise and signal amplitude (SNR). Here, SNR in any of the tested tunnels did not increase more than 1.3-fold, reaching a value of 10. Although the increase was statistically significant, the median SNRs were rather modest in comparison to those reported earlier for the much smaller, axon-encompassing microtunnels, where SNR has reached as high as 80 (Wang et al., 2012) or even 450 (Pan et al., 2014) depending on the tunnel dimensions. Furthermore, the SNR in the current study did not depend on tunnel dimensions. Thus, it seems likely that the slight improvement in SNR occurred because the PDMS device guided the neurons to grow on or near the electrodes, and shortened the distance between the signal source and detector (Obien et al., 2015). It is also possible that the cleaning procedures between the experiments have affected the signal detection properties of the MEAs. All and all, the results concur with earlier simulation studies and the general theory in the field (FitzGerald et al., 2008, 2009; Wang et al., 2012), which suggest that the current, comparatively high and wide, but short, tunnels do not provide high enough resistance to improve signal detection on MEA.

Although the tested PDMS tunnel devices had no major effects on SNR, we found that the tunnel devices dramatically increased the percentage of active electrodes and the amount of detected spike and burst activity on those electrodes when measuring hPSC-derived neuronal networks. Despite the advantages and potential of hPSC-derived networks in *in vitro* modelling (Hunsberger et al., 2015), they can be very slow (Odawara et al., 2016) and unpredictable in developing neuronal activity, and the low percentage of activity-detecting electrodes can especially complicate MEA data acquisition and analysis (Ylä-Outinen et al., 2010; Tukker et al., 2016). In our setup, the smallest of the tested tunnels were able to increase the portion of active electrodes up to 100% in only 3 weeks, increasing spike activity 12-fold and burst activity 30-fold at the same time. It is possible that the observed activity increases because the electrodes in the tunnels observe the same signal from multiple sites of the same neuron or network. However, in this case the signals in the tunnel electrodes would appear synchronous, and we observed synchronous activity only occasionally (spanning to two or three adjacent electrodes), while majority of the activity was non-synchronous. Increasing spike activity and temporal clustering of spikes into bursts are generally considered as signs of network activity maturation (Kapucu et al., 2012; Biffi et al., 2013; Odawara et al., 2016). It is possible that the maturation here was accelerated because the tunnels increased the density of neurons by limiting the area on which they can grow. However, according to earlier results from primary rodent cultures, simply increasing cell density cannot make the percentage of active electrodes exceed 60%, and once the maximum has been reached, increasing cell density merely decreases activity (Biffi et al., 2013). According to our own experience the maximum of active electrodes in hPSC-derived cultures is ~50%. This suggest that some additional feature of the smallest tunnels, such as the effect on neurite orientation or efficient placing of the network on the electrodes, can significantly increase the detected network activity.

Not only the smallest but also larger tunnels significantly increased the amount of detected activity in comparison to control MEAs, and in particular the electrodes outside the actual tunnels but under the PDMS lid detected very high spike and burst activity. This finding implies that either the outer walls of the tunnels (at a right angle to the tunnel itself, Figure 1) function as guiding barriers that localize the developing networks on the outside electrodes, or that the tunnels funnel the networks from the outside area (Figure 3A), or both. However, we cannot exclude the possibility that the increase in detected activity reflects an increase in sensitivity of the system because the extracellular signals are detected when they cross a threshold, which depends on the noise level. This phenomenon could explain the increased activity in the *h* = 43 μm devices in comparison to the *h* = 105 μm devices. Besides changes in measurement sensitivity, the differences in results between the tunnel heights can be influenced by the batch-to-batch variation of hPSCs. Still, the increased activity inside the tunnels followed the observed growth of the neuronal network, and differences in activity between the controls and PDMS device electrodes increased over time, indicating a facilitation of network activity development. The results suggest that PDMS tunnel devices are a valuable tool for increasing the measurable activity on MEA and can therefore save time and money when using hPSC-derived neurons in disease modelling, pharmacological testing or toxicological screening *in vitro*.

If tunnel devices are to be used for *in vitro* modelling, the activity inside the tunnels must be amenable to pharmacological manipulation. To date, reports from pharmacological experiments in tunnel devices are lacking, except one (Dworak and Wheeler, 2009). Here, we evaluated the effects of a well-established blocker of neuronal activity (TTX) on the MEA signals from entire neuronal networks inside PDMS tunnels. The MEA measurements were performed directly after addition of TTX to the medium, and the results showed that the pharmacological effect of a neuron-specific toxicant inside the tunnels was clear and manifested immediately.

We conclude that PDMS tunnel devices on MEAs are a suitable environment for cultivating neuronal networks of human origin. Although the effects of the tunnel devices on SNR were modest, the devices substantially increased the percentage of active electrodes and amount of detected neuronal network activity in a design-dependent and robust fashion. The tunnels are also suitable for pharmacological testing. Therefore, PDMS tunnel devices encompassing neuronal networks are a promising tool for reducing time and costs in analyses of the activity of hPSC-derived neuronal models on MEAs.

## Author contributions

PK, MR, and SN supervised the project; PK, MR, SN, LY, and MM designed experiments; MT performed experiments; MM assisted with the pharmacological experiments; LS produced the PDMS devices; MM, MT, and AP analysed data; MT and AP drafted the manuscript; MT, AP, MM, LY, LS, PK, MR, and SN revised the manuscript.

### Conflict of interest statement

The authors declares that the research was conducted in the absence of any commercial or financial relationships that could be construed as a potential conflict of interest.
