# Supplementary material for: Optimised PDMS Tunnel Devices on MEAs Increase the Probability of Detecting Electrical Activity from Human Stem Cell-Derived Neuronal Networks
Source: Front Neurosci. 2017 Oct 31;11:606. doi: 10.3389/fnins.2017.00606 (PMC5671636; doi:10.3389/fnins.2017.00606)
Supplement: Supplementary file 1 [file Presentation1.PDF]

Supplementary Material

PDMS Tunnel Devices on MEAs Increase the Measurable Activity of Human Stem Cell-Derived Neuronal Networks

Maria Toivanen, Anssi Pelkonen, Meeri Mäkinen, Laura Ylä-Outinen, Lassi Sukki, Pasi Kallio, Mervi Ristola*, Susanna Narkilahti*

*** Correspondence:** Mervi Ristola, mervi.ristola@uta.fi; Susanna Narkilahti, susanna.narkilahti@uta.fi

1 Supplementary Figures and Tables

1.1 Supplementary Figures

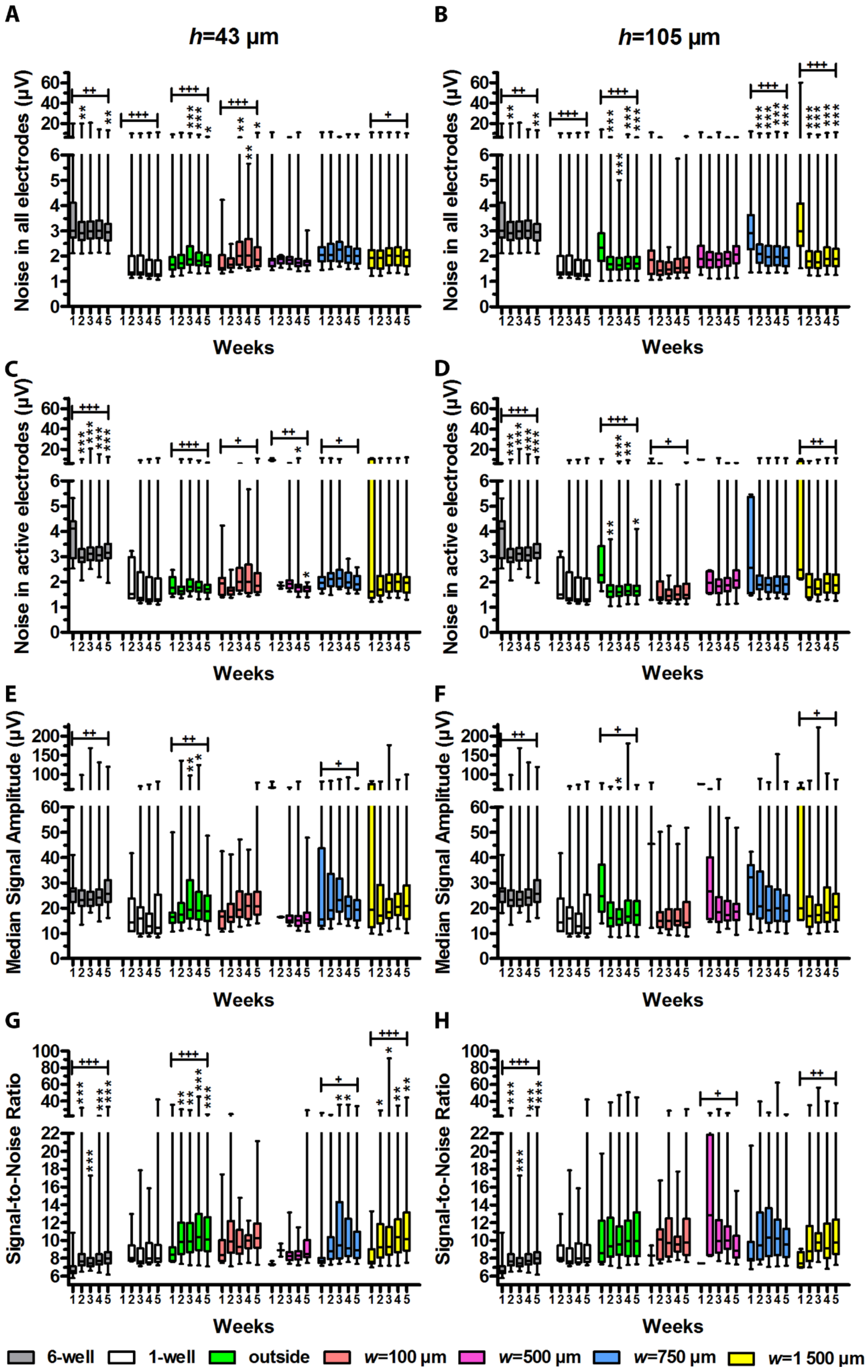
A
h=43 µm
B
h=105 µm
Noise in all electrodes (µV)
Weeks
C
D
Noise in active electrodes (µV)
Weeks
E
F
Median Signal Amplitude (µV)
Weeks
G
H
Signal-to-Noise Ratio
Weeks
6-well
1-well
outside
w=100 µm
w=500 µm
w=750 µm
w=1 500 µm

Supplementary Figure 1. Weekly noise in all and in active electrodes, signal amplitude and signal-to-noise ratio. The weekly noise was calculated for all electrodes in *h*=43 µm **(A)** and *h*=105 µm devices **(B)** and controls (6- and 1-well MEAs). Total *n* of analysed electrodes was 23-270 **(Table 1)**. The weekly noise was also calculated for the active electrodes in *h*=43 µm **(C)** and *h*=105 µm devices **(D)** and controls. Below these is presented the weekly median signal amplitude in active electrodes in *h*=43 µm **(E)** and *h*=105 µm devices **(F)** and controls. The weekly signal-to-noise-ratio in *h*=43 µm **(G)** and *h*=105 µm devices **(H)** and controls was calculated from the noise and median signal amplitude in active electrodes. *n* (active electrodes) was 1-167 **(Table 1)**. The + symbols indicate whether measurement week affected the results according to Kruskal-Wallis test (+: 0.05>p≥0.01; ++: 0.01 >p≥ 0.001; +++: p>0.001). The * symbols indicate the significance of the difference between the particular week and week 1 within the particular tunnel device or control according to Dunn's *post hoc* test (*: 0.05>p≥0.01; **: 0.01 >p≥ 0.001; ***: p>0.001).
